# Magnitude and trends of *Mycobacterium tuberculosis*: a retrospective cross-sectional study at Hargeisa TB Hospital, Hargeisa, Somaliland

**DOI:** 10.11604/pamj.2025.52.178.49142

**Published:** 2025-12-23

**Authors:** Ahmed Ibrahim Farah, Abdella Gemechu, Jama Mohamed, Naima Ahmed Mohmed, Aidarus Abdi Nur, Berhanu Seyoum

**Affiliations:** 1 College of Medicine and Health Sciences, University of Hargeisa, Hargeisa, Somaliland,; 2 National TB Program, Ministry of Health Development of Somaliland, Hargeisa, Somaliland,; 3 Haramaya University, College of Health and Medical Sciences, Harar, Ethiopia,; 4 Faculty of Statistics and Data Science, University of Hargeisa, Hargeisa, Somaliland,; 5 Doodi Hospital, Hargeisa, Somaliland,; 6 Hargeisa TB Hospital, Medical Laboratory Department, Hargeisa, Somaliland,; 7 Armauer Hansen Research Institute, Addis Ababa, Ethiopia

**Keywords:** *Mycobacterium tuberculosis*, GeneXpert MTB/Rif assay, MTB Rif resistance, Hargeisa TB Hospital, presumptive tuberculosis clients

## Abstract

**Introduction:**

tuberculosis (TB) is among the world´s top 10 infectious diseases, causing 1.5 million deaths annually, mostly in low-income countries. This study aimed to evaluate the magnitude and trends of Mycobacterium tuberculosis in Hargeisa, Somaliland.

**Methods:**

data from 18,280 presumptive TB clients enrolled between January 2020 and December 2023 at Hargeisa TB Hospital were analyzed using the GeneXpert Mycobacterium tuberculosis resistant to rifampicin assay. Data were entered into Excel, analyzed with SPSS v27, and assessed using Chi-square tests. Logistic regression was performed to estimate Adjusted Odds Ratios (AOR) with 95% Confidence Intervals (CI).

**Results:**

a total of 18,280 presumptive tuberculosis (TB) patients were included, with a mean age of 38.7 ± 20.2 years. Males accounted for 62.5% of the study population. Overall, 1,614 patients (8.8%) tested positive for TB. TB prevalence was higher in males (6.2%) than in females (2.6%), with males having greater odds of detection (adjusted odds ratio (AOR) 1.55; 95% CI: 1.38-1.73; p<0.001). Rifampicin resistance was identified in 43 cases (2.7%), more frequently in males (1.7%) than females (1.0%). TB detection declined from 10.2% in 2020 to 7.2% in 2023. The likelihood of rifampicin resistance was significantly higher in 2021-2023 compared with 2020 (AOR 9.29; 95% CI: 2.10-41.06; p<0.001 for 2021).

**Conclusion:**

the study revealed a significant burden of tuberculosis (TB) among presumptive pulmonary cases, with rifampicin resistance observed among confirmed patients.

## Introduction

Tuberculosis (TB) remains one of the top ten infectious diseases worldwide and the leading cause of death from a single pathogen. According to the World Health Organization (WHO), about one-third of the global population is infected with TB, with 5.8 million new cases and 1.5 million deaths reported in 2021. Approximately 95% of TB-related deaths occur in underdeveloped countries. In Somalia, TB is a major public health concern. The 2021 WHO report indicated that TB incidence in Somalia increased from 258 per 100,000 population in 2018 to 259 per 100,000 in 2020, with a death rate of 68 per 100,000 population [1].

The causative agent, *Mycobacterium tuberculosis*, is an obligate airborne bacterium that thrives in oxygen-rich areas of the body. Transmission occurs through coughing, sneezing, or close contact with an infected person [1,2]. Social and economic factors significantly influence TB epidemiology, complicating prevention, diagnosis, and treatment [3]. Early detection and appropriate therapy can prevent many TB-related complications, but disease management is often challenging.

On average, a bacteriologically confirmed TB patient can infect 10-15 people annually if untreated. In 2018, 3.4% of newly diagnosed and 18% of previously treated TB patients worldwide were infected with MDR-TB strains, complicating treatment and increasing costs [4]. The global TB control efforts are further threatened by drug-resistant strains. Multidrug-resistant (MDR) and extensively drug-resistant TB (XDR-TB) pose significant public health risks, undermining national TB programs and challenging the WHO “End TB by 2035” strategy [5,6]. Continuous monitoring through drug susceptibility testing (DST) is crucial to identify resistant strains in high-incidence regions [7].

Although only 14% of the global population lives in the WHO African region, it accounts for 25% of TB cases and the highest rates of HIV-associated TB comorbidity and mortality. Sub-Saharan Africa bears the greatest TB burden relative to population [8]. Sub-Saharan Africa is thought to bear 79% of the burden of HIV-associated tuberculosis (TB), with over 1.3 million cases and approximately 500,000 fatalities worldwide each year. Early in the HIV pandemic, TB mortality also rose in regions with high HIV and TB rates, especially in Africa and Asia [9].

Approximately 30-40% of TB cases remain undetected or unconfirmed for extended periods, highlighting the need for targeted interventions in high-risk populations, including people living with HIV and close contacts of TB patients [10]. This study aimed to determine the prevalence and trends of tuberculosis (TB) and multidrug-resistant TB (MDR-TB) among presumptive TB patients who visited Hargeisa TB Hospital, Somaliland, from 2020 to 2023.

## Methods

**Study design and setting:** this study was a retrospective cross-sectional study conducted at Hargeisa TB Hospital, the main TB referral center in Hargeisa, Marodi-Jeex District, Somaliland. The hospital, established in 1946, serves residents of Hargeisa and surrounding areas, a population of approximately 1.2 million. Hargeisa is located at 9.5612° N latitude and 44.0669° E longitude, at an elevation of 1,334 m above sea level. Data were collected from hospital records from 1^st^ January 2020 to 31^st^ December 2023.

**Study population:** the target population included all presumptive pulmonary TB patients who attended Hargeisa TB Hospital from 1^st^ January 2020 to 31^st^ December 2023. Inclusion criteria comprised patients with complete laboratory records in the GeneXpert TB registration log, while cases with incomplete or invalid/erroneous GeneXpert results were excluded. A total of 18,280 presumptive TB cases meeting these criteria were included in the study, representing a total population sample of all eligible patients during the study period.

**Data collection:** data were collected from the GeneXpert TB registration logbook, including patient demographics (age and sex), year of diagnosis, and laboratory results. Sputum samples from presumptive TB patients were collected and processed using the GeneXpert MTB/RIF Ultra system (Cepheid) according to the manufacturer´s instructions. Samples were decontaminated with a 2: 1 mixture of sodium hydroxide and isopropanol, transferred into test cartridges, scanned, and analyzed by the GeneXpert machine, generating results within two hours as ‘detected,´ ‘not detected´, or ‘invalid/error´ for *Mycobacterium tuberculosis* [11,12]. Since GeneXpert MTB/RIF can identify both *Mycobacterium tuberculosis* and rifampicin resistance, the WHO recommended it as the first diagnostic test for suspected HIV-associated or MDR-TB in high-incidence countries in 2010. Later, these recommendations were extended to all suspected TB cases while taking low-resource settings' resource constraints into account [13]. Finally, all relevant data were extracted from the register and entered into the study database for analysis.

**Definitions:** pulmonary tuberculosis (TB) was defined as the detection of *Mycobacterium tuberculosis* in sputum samples using the GeneXpert MTB/RIF Ultra assay. Multidrug-resistant TB (MDR-TB) was defined as TB exhibiting resistance to at least rifampicin, as determined by GeneXpert testing. The independent variables comprised age in years; sex, as male or female; and year of detection, corresponding to the year in which the diagnostic test was performed.

**Statistical analysis:** data were analyzed using SPSS version 27.0. Binary logistic regression was performed to assess the association between independent variables (age, sex, year of detection) and the outcomes (TB and MDR-TB). Crude odds ratios (CORs) were calculated for each variable, and all variables were included simultaneously in the multivariable model to obtain adjusted odds ratios (AORs) with 95% confidence intervals (CIs), adjusting for potential confounders. Statistical significance was set at p<0.05. The final model was interpreted to identify demographic factors independently associated with TB and MDR-TB detection.

**Ethical considerations:** ethical approval for this study was obtained from the National Ethical Committee of the Ministry of Health Development of Somaliland (reference: TIX: WHC/WX/3: 261). As this was a retrospective study using existing hospital records, individual informed consent was waived. All data were fully anonymized prior to extraction and analysis to ensure the confidentiality and privacy of participants.

## Results

**Characteristics of patients that visited Hargeisa TB Hospital from 2020-2023, Hargeisa, Somaliland (N=18,280):** between 2020 and 2023, a total of 18,280 presumptive TB patients were screened for *Mycobacterium tuberculosis* at Hargeisa TB Hospital. The characteristics of the study population are summarized in Table 1. Males accounted for 62.5% (11,418) of the participants, while females constituted 37.5% (6,862). The age distribution (mean ± SD = 38.69 ± 20.247) indicates that the largest proportion of patients, 30.7% (5,607), were between 11 and 25 years old, followed by 25.7% (4,699) in the 26-40 age group. Children under 10 years accounted for 3.7%, whereas 6.3% were aged 71 years and above. Over the four-year period, the number of patients screened increased steadily, with the highest number recorded in 2023 (5,926; 32.4%), compared to 4,044 (22.1%) in 2020. Overall, the study population demonstrated a predominance of males and a progressive increase in screening volume across the study years.

**Table 1:** characteristics of patients who visit Hargeisa TB Hospital from 2020-2023, Hargeisa, Somaliland (N=18,280)

	Frequency	Percent (%)	Total (%)
**Sex**			
Female	6862	37.5	6862
Male	11418	62.5	11418
			18280 (100%)
**Age-groups**			
Under 10	682	3.7	682
11-25	5607	30.7	5607
26-40	4699	25.7	4699
41-55	3070	16.8	3070
56-70	3065	16.8	3056
71 and older	1157	6.3	1157
			18280 (100%)
**Year**			
2020	4044	22.1	4044
2021	3782	20.7	3782
2022	4525	24.8	4525
2023	5926	32.4	5925
			18280 (100%)

**Figure 1:**
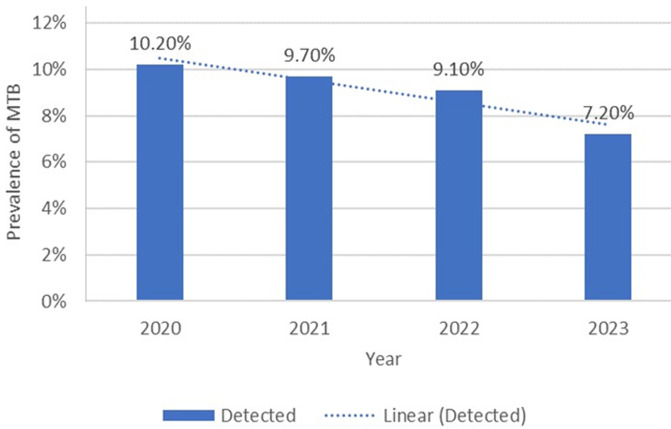
trends of *Mycobacterium tuberculosis* prevalence in the screened population in Hargeisa, Somaliland, from 2020-2023

**Trends of *Mycobacterium tuberculosis* prevalence in the screened population in Hargeisa, Somaliland, from 2020-2023:** furthermore, we investigated the trends of *Mycobacterium tuberculosis* prevalence in the screened population from 2020-2023. These trends can be seen in Figure 1. The data indicates the percentage of detected cases shows a gradual decrease from 10.2% in 2020 to 7.2% in 2023. This trend is highlighted by the dotted linear lines for detected cases, showing a slight downward trend. This suggests a decrease in the prevalence of *Mycobacterium tuberculosis* in the screened population over this period. However, this still needs further public health interventions to continue and potentially accelerate the downward trend in *Mycobacterium tuberculosis* prevalence.

***Mycobacterium tuberculosis* status in the screened population in Hargeisa, Somaliland from 2020-2023 (bivariate analysis):** to identify the association between sex, age groups, and years with MTB status and MTB rifampicin resistance status, we calculated the Crude Odds Ratio (COR) and Adjusted Odds Ratio (AOR) using binary logistic regression. Table 2 summarizes the *Mycobacterium tuberculosis* (MTB) status among the screened population at Hargeisa TB Hospital from 2020 to 2023, examining associations with sex, age group, and year of screening. Among the total population screened, 6.2% were males with MTB detected, while 2.6% were females with MTB detected, indicating a significantly higher likelihood of detection in males (AOR: 1.550, 95% CI: 1.384-1.734, p<0.001).

**Table 2:** *Mycobacterium tuberculosis* status in the screened population in Hargeisa, Somaliland from 2020-2023 (bivariate analysis)

Variable	MTB-status		χ^2^ (P-Value)	COR (95% CI)	P-value	AOR (95% CI)	P-value
	Detected (N/%)	Not detected (N/%)					
**Sex**			**<.001**				
Female	474(2.6%)	6388 (34.9%)		1.000 (Ref)		1.000 (Ref)	
Male	1140(6.2%)	10278 (56.2)		1.495 (1.337, 1.671)	<0.001	1.550 (1.384, 1.734)	<0.001
**Age-group**			**<.001**		**<0.001**		**<0.01**
Under 10 yrs	26 (0.1%)	656 (3.6%)		1.000 (Ref)		1.000 (Ref)	
11-25	729 (4.0%)	4878 (26.9%)		3.771 (2.529, 5.623)	<0.001	3.592 (2.407, 5.361)	<0.001
26-40	476 (2.6%)	4223 (23.1%)		2.844 (1.900, 4.256)	<0.001	2.658 (1.774, 3.982)	<0.001
41-55	209 (1.1%)	2861 (15.7%)		1.843 (1.215, 2.795)	0.004	1.703 (1.122, 2.585)	0.012
56-70	131 (0.7%)	2934 (16.1%)		1.127 (0.733, 1.730)	0.586	1.045 (0.680, 1.607)	0.841
71 and older	43 (0.2%)	1114 (6.1%)		0.974 (0.593, 1.600)	0.917	0.880 (0.535, 1.447)	0.615
**Year**			**<.001**		**<0.001**		**<0.001**
2020	411 (2.2%)	3633 (19.9%)		1.000 (Ref)		1.000 (Ref)	
2021	365 (2.0%)	3417 (18.7%)		0.944 (0.814, 1.095)	0.449	0.968 (0.833, 1.125)	0.669
2022	410 (2.2%)	4115 (22.5%)		0.881 (0.763, 1.017)	0.084	0.893 (0.772, 1.033)	0.129
2023	428 (2.3%)	5501 (30.1%)		0.688 (0.597, 0.792)	<0.001	0.691 (0.598, 0.797)	<0.001

MTB: *Mycobacterium tuberculosis*; COR: crude odds ratio; AOR: adjusted odds ratio; CI: confidence interval

Regarding age, 4.0% of those screened were in the 11-25 age group with MTB detected, showing the highest detection rate compared to 0.1% among those under 10 years old (AOR: 3.592, 95% CI: 2.407-5.361, p<0.001). Across the four years, 2.3% of the total screened population had MTB detected in 2023, compared to 2.2% in 2020, though the likelihood of MTB detection was slightly lower in 2023 (AOR: 0.691, 95% CI: 0.598-0.797, p<0.001). This data highlights a greater proportion of MTB detection among males and the 11-25 age group, with a slight decline in detection rates over the study period.

***Mycobacterium tuberculosis* rifampicin resistance status in the screened population in Hargeisa, Somaliland from 2020-2023 (multivariate analysis):** Table 3 presents the *Mycobacterium tuberculosis* rifampicin (MTB-Rif) resistance status among the screened population at Hargeisa TB Hospital from 2020 to 2023, analyzing associations with sex, age group, and year of screening. Among the total screened population, 1.7% were males with MTB-Rif resistance detected, while 1.0% were females, though the difference was not statistically significant (AOR: 0.678, 95% CI: 0.359-1.281, p=0.255). For age groups, 1.1% of the screened population aged 11-25 and 26-40 had MTB-Rif resistance, with the highest proportion in this age range, although no significant differences were observed compared to other age groups. This still justifies further investigation and potentially enhanced public health measures focused on this demographic to control and prevent the spread of MTB-Rif resistance. The yearly breakdown shows that the proportion of resistance was relatively consistent, with the highest in 2021 and 2023 at 0.9% and the lowest in 2020 at 0.1%. However, the likelihood of resistance was significantly higher in 2021, 2022, and 2023 compared to 2020 (AOR: 9.294, 95% CI: 2.104-41.059, p<0.001 for 2021). This data suggests that while MTB-Rif resistance was detected in both males and females, the most notable finding is the significantly higher likelihood of resistance in later years compared to 2020.

**Table 3:** *Mycobacterium tuberculosis* rifampicin resistance status in the screened population in Hargeisa, Somaliland from 2020-2023 (multivariate analysis)

Variable	MTB-Rif status		χ^2^ (P-value)	COR (95% CI)	P-value	AOR (95% CI)	P-value
	**Resistant (N/%)**	**Susceptible (N/%)**					
**Sex**			**0.255**				
Female	16 (1.0%)	458 (28.4%)		1.000 (Ref)		1.000 (Ref)	
Male	27 (1.70%)	1111 (68.9%)		0.696 (0.371, 1.303)	0.257	0.678 (0.359, 1.281)	0.231
**Age-group**			**0.303**		**0.495**		**0.404**
Under 10 yrs	2 (0.1%)	24 (1.5%)		1.000 (Ref)		1.000 (Ref)	
11-25	17 (1.1%)	711 (44.1%)		0.287 (0.063, 1.313)	0.108	0.266 (0.057, 1.246)	0.093
26-40	17 (1.1%)	459 (28.5%)		0.444 (0.097, 2.035)	0.296	0.438 (0.093, 2.067)	0.297
41-55	4 (0.2%)	205 (12.5%)		0.234 (0.041, 1.346)	0.104	0.221 (0.037, 1.311)	0.097
56-70	3 (0.2%)	127 (7.9%)		0.283 (0.045, 1.788)	0.180	0.250 (0.038, 1.629)	0.147
71 and older	0 (0.0%)	43 (2.7%)		-	0.998	-	0.998
**Year**			**0.009**		**0.034**		**0.029**
2020	2 (0.1%)	407 (25.2%)		1.000 (Ref)		1.000 (Ref)	
2021	15 (0.9%)	350 (21.7%)		8.721 (1.981, 38.401)	0.004	9.294 (2.104, 41.059)	0.003
2022	11 (0.7%)	399 (24.8%)		5.610 (1.236, 25.471)	0.025	5.891 (1.294, 26.817)	0.022
2023	15 (0.9%)	413 (25.6%)		7.391 (1.680, 32.525)	0.008	7.498 (1.700, 33.073)	0.008

MTB-Rif: *Mycobacterium tuberculosis* rifampicin resistant; COR: crude odds ratio; AOR: adjusted odds ratio; CI: confidence interval

## Discussion

This study aimed to determine the magnitude and trends of *Mycobacterium tuberculosis* and rifampicin-resistant TB among presumptive TB patients attending Hargeisa TB Hospital between 2020 and 2023. Overall, 8.83% of the screened patients tested positive for TB, and 2.7% of these cases were rifampicin-resistant. TB was more prevalent among males, and the most affected age groups were 11-25 and 26-40 years. Over the study period, there was a steady increase in the number of patients screened. These findings provide a comprehensive overview of the current burden and trends of TB in Hargeisa.

In the current study, 8.83% of the total screened individuals tested positive for TB using GeneXpert, which has been shown to have high specificity and sensitivity for TB detection [14]. The proportion of *Mycobacterium tuberculosis* infection observed in this study was lower than that reported in studies conducted at Mizan-Tepi University, Southwest Ethiopia (12.5%) [12], among displaced populations residing in refugee camps in Ethiopia (13.3%) [15], and in other studies reporting prevalences of 12.2% and 11.2%, respectively [16,17]. However, our findings showed a higher proportion of TB detection compared to adults attending Finchwa Health Center in West Guji Zone, Southern Ethiopia (4.8%) [18]. Similarly, the prevalence observed here was lower than that reported at Nyamira County Referral Hospital in Kenya (26.69%) [19], in Nigeria (22.9%) [20], and in Sudan (26.6%) [21]. These variations may be attributable to differences in climate, geography, diagnostic techniques, and study populations.

Regarding rifampicin resistance, 47 patients (2.7%) in this study were identified as resistant. This prevalence was lower than that reported in Kenya (4.6%) [22], Ethiopia (9%) [23], and Zimbabwe (4.5%) [24]. Rifampicin resistance was higher among males (1.7%) than females (1.0%), consistent with findings from Nigeria, which reported 9.1% in males and 4.4% in females [25], although a study in Ethiopia found higher resistance among females [26]. These differences may reflect variations in TB frequency across populations, sampling methods, study settings, and diagnostic techniques. Age-wise, the highest frequency of rifampicin-resistant TB was observed among individuals aged 11-25 and 26-40 years, each representing 1.1% (2.2%) of cases. This aligns with studies reporting the highest frequency of MDR-TB among individuals aged 15-39 years [26] and, in some studies, higher rates among children under 15 [27]. Differences in findings could be explained by population characteristics, sample sizes, and methodologies.

Males were significantly more likely to be diagnosed with TB than females, consistent with studies conducted in Sudan [21], Nepal [27], Pakistan [28], and Ethiopia [29]. Behavioral and environmental factors, including smoking and chewing khat in poorly ventilated rooms, which are common in regions such as Somaliland, may contribute to higher male TB cases [30,31]. The most affected age groups in this study were 11-25 and 26-40 years. This is similar to findings in Ethiopia, where individuals over 15 years had the highest TB prevalence [32], but differs from studies in Kenya reporting the highest frequency among children under 15 years [33]. These discrepancies may be due to differences in study populations and methodologies.

This study is significant for several reasons. Firstly, it is the first report on the magnitude and trends of *Mycobacterium tuberculosis* in Hargeisa, Somaliland. Secondly, it included a large number of presumptive TB clients over a four-year period from a single TB hospital (Hargeisa TB Hospital), providing comprehensive insight into TB trends in the region. Thirdly, the use of GeneXpert MTB/RIF as a diagnostic tool in this context represents the first application of this assay for TB detection in Hargeisa. These findings highlight the importance of continued surveillance, early detection, and targeted interventions, and they provide a foundation for future studies on TB in the region.

The study has certain limitations. The use of retrospective record analysis meant that some sociodemographic information, such as residence, employment, and educational background, was not available. Clinical data that could have enhanced the analysis were also limited. Additionally, the study relied solely on GeneXpert MTB/RIF results without comparison to other diagnostic methods, such as smear microscopy or culture, which may affect the comprehensiveness of the findings. Despite these limitations, the study has several strengths, including the large sample size over multiple years, the focus on a single TB hospital, which provides consistent data collection, and the use of a highly specific and sensitive diagnostic assay, which enhances the reliability of TB detection in this population.

## Conclusion

This study demonstrated a notable burden of tuberculosis (TB) and rifampicin-resistant *Mycobacterium tuberculosis* (MTB-Rif) in Somaliland. Males and individuals under 40 years were more likely to present with rifampicin-resistant TB. The trends observed between 2020 and 2023 indicate an increasing detection of resistant cases over time. These findings highlight the need for strengthened TB control measures, including preventive strategies, effective management of new cases, regular drug resistance surveillance, and rigorous implementation of infection prevention and control practices. Addressing these areas is critical to reducing the spread of TB and rifampicin-resistant strains, improving patient outcomes, and informing public health interventions in Somaliland.

### What is known about this topic

- Tuberculosis (TB) remains a leading cause of death worldwide and one of the top ten causes of mortality;
- Sub-Saharan Africa carries a high TB burden with ongoing challenges in diagnosis and control;
- GeneXpert MTB/RIF is widely used in developing countries, improving the detection of TB and rifampicin resistance.

### What this study adds

- Provides updated evidence on TB magnitude and trends in Hargeisa using 18,280 presumptive patient records;
- Reports a TB positivity rate of 8.8%, offering baseline data for program planning and evaluation;
- Demonstrates the utility of GeneXpert and routine logbook data for monitoring TB and guiding public health interventions.
